# Assessment of myocardial strain using strain-encoding (SENC) MRI: comparison of acquisition strategies

**DOI:** 10.1186/1532-429X-13-S1-P15

**Published:** 2011-02-02

**Authors:** El-Sayed H Ibrahim, Wolfgang Rehwald, Bradley P Sutton, Sven Zuehlsdorff, Richard D White

**Affiliations:** 1Department of Radiology, University of Florida, Jacksonville, FL, USA; 2Siemens Medical Solutions, MRI Cardiovascular R&D, Chicago, IL, USA; 3Department of Bioengineering, University of Illinois, Urbana-Champaign, IL, USA

## Introduction

**Figure 1 F1:**
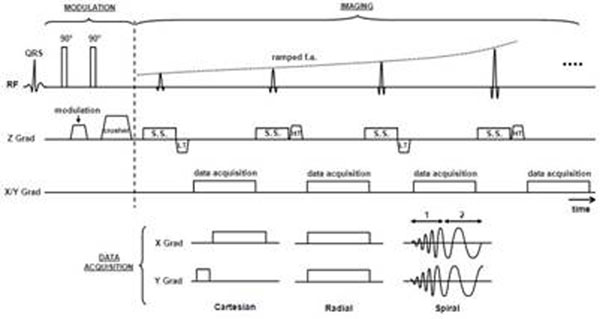
SENC pulse sequence. The pulse sequence consists of two parts: modulation and imaging. The modulation part is composed of two non-selective 90° RF pulses, interspersed by a modulation gradient in the slice-selection direction, and followed by a crusher gradient. The imaging part is composed of a series of ramped slice-selective RF pulses, each followed by a demodulation (tuning) gradient in the slice-selection direction, and then data acquisition. Interleaved tunings were implemented to reduce scan time. Data acquisition can be either Cartesian, Radial or Sprial, as shown in the bottom. Spiral gradients were designed to consist of two parts: 1) slew-rate limited and 2) amplitude limited.

Strain-encoding (SENC) MRI was recently introduced for measuring strain with high-resolution and simple post-processing(1). Figure-1 shows SENC pulse sequence. In typical SENC sequence, k-space data is acquired line-by-line in rectilinear fashion, which results in long scan-time and renders the technique impractical for many applications. Nevertheless, fast imaging techniques, e.g. Radial or Spiral acquisition, allow for reducing scan time while maintaining adequate image quality. In this work, Radial and Spiral acquisitions were implemented in SENC for improved performance. The developed sequences were tested on volunteers and the results were evaluated and compared to standard Cartesian acquisition.

## Methods

Radial and Spiral acquisitions(2) were implemented in SENC, and the different sequences were tested on three volunteers on Siemens Tim-Trio 3.0-Tesla scanner. The imaging parameters were: FOV=350mm, slice-thickness =10mm, flip-angle=15°, # heart-phases=25, and scan time=17 s. The three sequences were optimized for the fixed scan-time. Maximum resolution obtained was 160x160 for Cartesian (80% phase-encoding coverage) and Radial (128 Radial spokes), and 256x256 for Spiral (10 spiralsx2 averages). The low-tuning and high-tuning images were combined together as described in(1) to construct the strain images. Strain values were measured at five different points along the lateral left-ventricular wall on all volunteers. Bland-Altman analysis was conducted to compare measurements from different sequences.

## Results

**Figure 2 F2:**
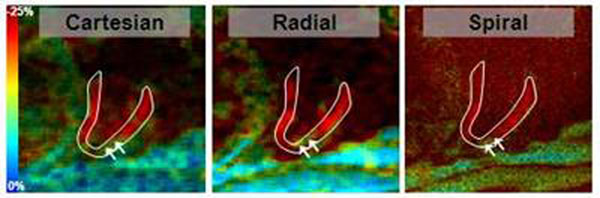
SENC two-chamber images from Cartesian (left), Radial (middle), and Spiral (right) acquisitions showing circumferential strain at end-systole. Carteseian and Radial show similar image quality, while Spiral shows superior resolution. All images show similar contracting pattern in the heart. The arrows point to part of the apical wall showing low strain, which showed in all images.

**Figure 3 F3:**
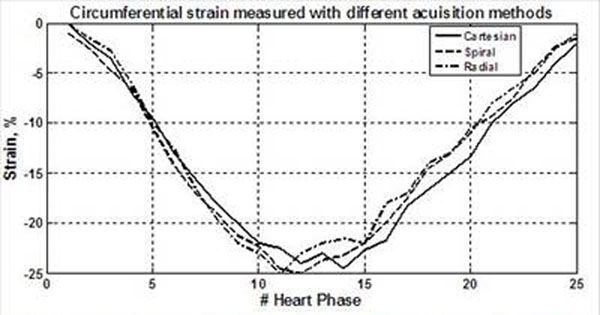
Circumferential strain curves through the cardiac cycle (25 phases) measured from SENC images with Cartesian (solid), dashed (Spiral), and Radial (dot-dashed) acquisitions. The curves show similar contracting pattern irrespective of the acquisition strategy.

**Figure 4 F4:**
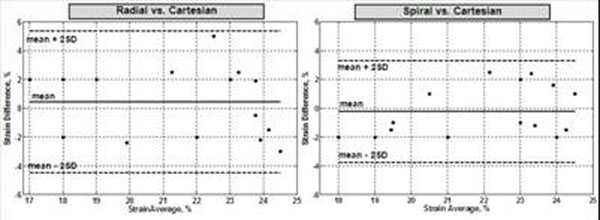
Bland-Altman plots for the correlation between strain measurements from SEND images with different acquisition strategies. Radial vs. Cartesian (left) and Spiral vs. Cartesian (right). The plots show good agreement between the different techniques, despite more variabilities with Radial acquisition. All measurement differences lied within the ±2SD limits.

Figure-2 shows example of the acquired SENC images. The strain values measured at the same position were similar in different images, as shown in the strain curves in Figure-3. The Bland-Altman analysis showed no bias between strain measurements from different acquisitions (Figure-4). The mean±SD of the (circumferential) strain differences were 0.42±2.46 and -0.25±1.76 % for the Radial-Cartesian and Spiral-Cartesian differences, respectively. All the differences lied within the ±2SD limit.

## Discussion and conclusions

Data acquisition strategy (k-space trajectory) affects scan-time and the resulting image-quality in SENC. Image quality was similar in Cartesian and Radial. Less radial spokes can be acquired to reduce scan-time without much affecting image-quality. Due to its acquisition nature, spatial-resolution is compromised in Radial acquisition. For the same scan time, Spiral acquisition allowed for improving resolution by more than 60% and doubling # averages, compared to Cartesian or Radial, despite longer reconstruction time. High spatial-resolution would allow for accurate measurements in small structures, e.g. thinning myocardial wall, or it can be traded for faster or real-time imaging. The choice of the acquisition-technique depends on patient condition, available scan-time, and imaging features of importance.

## References

OsmanMRM2001324334GloverGHMRM199941241510.1002/(sici)1522-2594(199908)42:2<412::aid-mrm25>3.0.co;2-u10440968
